# Plasma phosphorylated tau181 as a biomarker of mild traumatic brain injury: findings from THINC and NCAA-DoD CARE Consortium prospective cohorts

**DOI:** 10.3389/fneur.2023.1202967

**Published:** 2023-08-17

**Authors:** Christina Devoto, Rany Vorn, Sara Mithani, Timothy B. Meier, Chen Lai, Steven P. Broglio, Thomas McAllister, Christopher C. Giza, Daniel Huber, Jaroslaw Harezlak, Kenneth L. Cameron, Gerald McGinty, Jonathan Jackson, Kevin Guskiewicz, Jason P. Mihalik, Alison Brooks, Stefan Duma, Steven Rowson, Lindsay D. Nelson, Paul Pasquina, Christine Turtzo, Lawrence Latour, Michael A. McCrea, Jessica M. Gill

**Affiliations:** ^1^Henry M. Jackson Foundation for the Advancement of Military Medicine, Bethesda, MD, United States; ^2^National Institute of Neurological Disorders and Stroke, National Institutes of Health, Bethesda, MD, United States; ^3^School of Nursing, Johns Hopkins University, Baltimore, MD, United States; ^4^School of Nursing, University of Texas Health at San Antonio, San Antonio, TX, United States; ^5^Department of Neurosurgery, Medical College of Wisconsin, Milwaukee, WI, United States; ^6^Center for Neuroscience and Regenerative Medicine, Uniformed Services University and Health Science, Bethesda, MD, United States; ^7^Michigan Concussion Center, University of Michigan, Ann Arbor, MI, United States; ^8^Department of Psychiatry, Indiana University School of Medicine, Indianapolis, IN, United States; ^9^Departments of Pediatrics and Neurosurgery, UCLA Steve Tisch BrainSPORT Program, University of California, Los Angeles, Los Angeles, CA, United States; ^10^Department of Epidemiology and Biostatistics School of Public Health-Bloomington, Indiana University, Bloomington, IN, United States; ^11^John A. Feagin Sports Medicine Fellowship, Keller Army Hospital, West Point, NY, United States; ^12^United States Air Force Academy, Colorado Springs, CO, United States; ^13^Matthew Gfeller Center, Department of Exercise and Sport Science, University of North Carolina at Chapel Hill, Chapel Hill, NC, United States; ^14^Department of Orthopedics and Sports Medicine, University of Wisconsin, Madison, WI, United States; ^15^Department of Biomedical Engineering, Virginia Tech, Blacksburg, VA, United States; ^16^Department of Neurology, Johns Hopkins University, Baltimore, MD, United States

**Keywords:** brain trauma, p-tau181, mild traumatic brain injury, mTBI, sports related concussion, concussion

## Abstract

**Objective:**

The aim of this study was to investigate phosphorylated tau (p-tau181) protein in plasma in a cohort of mild traumatic brain injury (mTBI) patients and a cohort of concussed athletes.

**Methods:**

This pilot study comprised two independent cohorts. The first cohort—part of a Traumatic Head Injury Neuroimaging Classification (THINC) study—with a mean age of 46 years was composed of uninjured controls (UIC, *n* = 30) and mTBI patients (*n* = 288) recruited from the emergency department with clinical computed tomography (CT) and research magnetic resonance imaging (MRI) findings. The second cohort—with a mean age of 19 years—comprised 133 collegiate athletes with (*n* = 112) and without (*n* = 21) concussions. The participants enrolled in the second cohort were a part of a multicenter, prospective, case-control study conducted by the NCAA-DoD Concussion Assessment, Research and Education (CARE) Consortium at six CARE Advanced Research Core (ARC) sites between 2015 and 2019. Blood was collected within 48 h of injury for both cohorts. Plasma concentration (pg/ml) of p-tau181 was measured using the Single Molecule Array ultrasensitive assay.

**Results:**

Concentrations of plasma p-tau181 in both cohorts were significantly elevated compared to controls within 48 h of injury, with the highest concentrations of p-tau181 within 18 h of injury, with an area under the curve (AUC) of 0.690–0.748, respectively, in distinguishing mTBI patients and concussed athletes from controls. Among the mTBI patients, the levels of plasma p-tau181 were significantly higher in patients with positive neuroimaging (either CT+/MRI+, *n* = 74 or CT−/MRI+, *n* = 89) compared to mTBI patients with negative neuroimaging (CT−/MRI−, *n* = 111) findings and UIC (*P*-values < 0.05).

**Conclusion:**

These findings indicate that plasma p-tau181 concentrations likely relate to brain injury, with the highest levels in patients with neuroimaging evidence of injury. Future research is needed to replicate and validate this protein assay's performance as a possible early diagnostic biomarker for mTBI/concussions.

## Introduction

Traumatic brain injuries (TBIs), including mild traumatic brain injury (mTBI) or *concussion*, are common, with more than 2.87 million individuals seeking acute care for these injuries each year (1). Accurate diagnosis of TBI is critical to ensuring effective clinical intervention and risk stratification, particularly among those most vulnerable to repetitive injury and long-term neurological symptoms (2–4). Conventional diagnostic tools relied upon in the care of more severe injuries to confirm diagnosis and inform on pathology have limited sensitivity to more mild forms of injury, including sports-related concussions (SRC), which reflect the mildest form of mTBI. Consequently, mTBI/SRC diagnosis relies upon a detailed clinical exam that is often supported by a neuropsychological assessment and subjective symptom inventories, with the time of symptom resolution serving as a proxy indicator for injury resolution. In a recent advanced imaging study within the Concussion Assessment, Research, and Education (CARE) cohort, white matter abnormalities detected with diffusion-tensor imaging (DTI) were observed acutely and found to persist beyond the asymptomatic time point and up to 6 months post-injury (5). Acute axonal injury and microstructural alterations of white matter are hallmark features of mTBI, but their subtle nature can often only be detected in research settings with DTI and fractional anisotropy (FA). Since advanced imaging modalities such as DTI are not widely available in emergency room settings, alternative biomarkers sensitive to the subtle nature of these injuries are critically needed to inform clinical decisions. The lack of objective injury indicators is a significant barrier to improve mTBI diagnosis, outcomes, and effective clinical management, which are needed to mitigate the long-term neurological risks linked to mTBI, including neurodegenerative diseases (ND) (2). Blood-based biomarkers offer the potential for objective indicators of injury as well as insight into the neurodegenerative processes underlying the risk of ND linked to mTBI exposure. Phosphorylated tau (p-tau) is pathologically linked to Alzheimer's disease (AD), chronic traumatic encephalopathy (CTE), and other NDs associated with TBI and has recently emerged as a promising, potentially sensitive biomarker of acute mTBI (3, 6).

The axonal protein tau is a microtubule-associated protein. Post-translational modifications of tau are a pathogenic feature of tauopathies. Phosphorylation of tau in the CNS is common after TBI, with acute elevations of p-tau being detectable within hours post-injury (7, 8). Acute axonal injury associated with TBI results in the disruption of normal tau binding to tubulin, thereby exposing multiple phosphorylation sites (9). Unlike normal tau, abnormal p-tau is insoluble and, therefore, favors a paired helical filament arrangement that progressively aggregates to form the main components of neurofibrillary tangles (NFT)—a hallmark of tauopathies, including AD and CTE (4, 6, 10). Major pathogenic sites include threonine (Thr) 181 and Thr231 (11). P-tau and total-tau (t-tau) levels in the cerebral spinal fluid (CSF) are diagnostic markers of AD, but only recently has their detection in the blood been possible. P-tau181 has been found to be more strongly associated with markers of AD than t-tau (12–14). However, the clinical utility of plasma p-tau181 in TBI and mTBI, in particular, is unclear. Plasma p-tau181 has been shown to have diagnostic and prognostic utility for AD, regardless of the clinical stage (15, 16). It has recently been shown to correlate with CSF levels and cerebral amyloid-beta and tau pathology (17). Increased levels of plasma p-tau181 have been observed at the preclinical stage of AD and are further increased in mild cognitive impairment (MCI) and AD patients (15, 16). In asymptomatic patients and patients with MCI, increased plasma p-tau181 was shown to be predictive of future transition to AD (15). Acute and chronic elevations in total tau have been reported following mTBI in athletes with SRC and in military professionals with chronic post-concussive symptoms; however, recent studies suggest that p-tau and p-tau ratio may be more sensitive to mTBI than t-tau (18, 19). In a study of acute (mild-severe) TBI, p-tau231 and the p-tau–t-tau ratio were superior to t-tau as diagnostic and prognostic biomarkers of acute TBI (20). Plasma p-tau231 levels and the p-tau–t-tau ratio were elevated in patients with all severities of acute TBI compared with healthy controls and were also shown to distinguish CT+ vs. CT− scans among all patients with mTBI, whereas t-tau could not (20). In a study evaluating age-related differences in the diagnostic accuracy of plasma glial fibrillary acidic protein (GFAP), t-tau, p-tau231, and p-tau-ratio for identifying acute intracranial trauma among mTBI patients, p-tau231 concentrations showed the highest diagnostic accuracy in the pooled cohort and in age-stratified analyses for diagnosing intracranial trauma on CT (21). Recent studies have also shown serum p-tau231 to be elevated in clinical studies of severe TBI and in a rodent model of repetitive mTBI during both the acute and subacute periods (20, 22). However, the utility of p-tau181 in acute mTBI patients and concussed athletes is not yet known. In chronic military-related mTBI, exosomal t-tau and p-tau181 have been shown to be elevated in repetitive mTBI (23). In a study examining the proteomic profiles of brain-derived extracellular vesicles (EVs) separated from the brain tissue of CTE cases and an age-matched control group with no history of contact sports, CTE EVs were found to be enriched in p-tau181 and neuron-specific molecules (24). Additionally, the p-tau181 levels were shown to be significantly higher in CTE EVs compared to control EVs and could distinguish the two groups with 73.6% accuracy (24). P-tau181 in CSF-derived EVs has also recently been shown as a potential monitoring biomarker in former NFL players at risk for CTE (25). Plasma p-tau181 was also recently shown to be significantly higher in retired contact sport athletes compared to healthy controls and was significantly associated with MRI brain imaging abnormalities in the athlete group (26). In military-related TBI, elevations in serum p-tau181 levels were observed in the military personnel at 2 and 7 days following repetitive blast exposure (27).

The current study investigated the association of acute SRC with plasma p-tau181 levels in collegiate athletes compared to control athletes without concussion and with mTBI patients with and without neuroimaging findings (CT+/MRI+; CT−/MRI+; CT−/MRI−) compared to uninjured controls (UIC). To the best of our knowledge, this is the first study to investigate plasma p-tau181 in acute mTBI patients presenting at the emergency department and athletes with SRCs. We hypothesized that plasma p-tau181 levels would be elevated in (1) athletes with concussion compared to control athletes without concussion; (2) mTBI patients compared to UIC; and (3) mTBI patients with positive imaging findings (CT+/MRI+ or CT−/MRI+) compared with mTBI patients without imaging findings (CT−/MRI−) and UIC.

## Methods

### Participants

#### Traumatic Head Injury Neuroimaging Classification cohort

Participants were recruited as part of the Traumatic Head Injury Neuroimaging Classification (THINC) study (NCT01132937), which is a large and ongoing natural history investigation of TBI. Patients, who were aged between 18 and 96 years, seeking emergency care for suspected mild TBI were included within our analyses (*n* = 288). UIC without a history of TBI or neurological disease were recruited from the NIH protocols: NCT01762475 and 09-NR-0131. UIC matched on age, sex, race, and ethnicity were included for comparison (*n* = 30). Enrollment, blood collection, clinical computed tomography (CT) and research magnetic resonance imaging (MRI) were performed within 48 h of injury for all mTBI patients. Subanalyses were performed by stratifying participants by time from injury to blood sampling (0–6 h of injury, 7–18 h of injury, 19–24 h of injury, and >25 h of injury). Relevant protocols were approved by the respective institutional review boards, and informed consent was obtained prior to data collection. An initial Glasgow Coma Scale was performed in the emergency department to assess the severity of injury. The neurobehavioral symptom inventory (NSI) was used to assess the participants' symptom experiences following TBI. The NSI is a Likert scale with scores from 0 to 4 (0 = none, 1 = mild, 2 = moderate, 3 = severe, and 4 = very severe), and it is divided into the following four subscales: affective, cognitive, somatic, and somatosensory. Higher total scores indicated more severe symptoms after mTBI.

#### Concussion Assessment, Research, and Education (CARE) Consortium cohort

A total of 133 athletes with and without concussions were included in the analysis. Concussion athletes were defined according to the consensus definition from the US Department of Defense's evidence-based guidelines (28). Concussed athletes (*n* = 112) and same-sport non-concussed athletes (*n* = 21) were included for comparison, with all athletes having provided blood samples at the same post-injury (0–48 h) time point. Sub-analyses were performed by stratifying participants by time from injury to blood sampling (0–6 h of injury, 7–18 h of injury, 19–24 h of injury, and >25 h of injury). Non-concussed athlete controls were matched to concussed athletes on age, sex, race, ethnicity, institution, and sports injury mechanism. All the participants were administered the Sport Concussion Assessment Tool-Third Edition (SCAT-3) symptoms evaluation, the Standardized Assessment of Concussion (SAC), the Balance Error Scoring System (BESS), and the Brief Symptom Inventory 18 (BSI-18) at the same time point of blood collection.

Participants included in this study were a part of a larger cohort study conducted by the National Collegiate Athletic Association (NCAA)–Department of Defense Concussion CARE Consortium. The CARE Consortium was approved by the Medical College of Wisconsin Institutional Review Board and the Human Research Protection Office at the US Army Medical Research and Material Command, and written informed consent was obtained for all participants. The CARE Consortium Advanced Research Core has been detailed elsewhere (29). Briefly, the CARE Advanced Research Core post-injury protocol included a collection of clinical data and blood samples from athletes with or without concussions at the following time points: post-injury (0–48 h post-injury), asymptomatic post-injury (start of the return to play protocol), 7 days after unrestricted return to play, and 6 months post-injury. Clinical assessments were performed at all time points and included the SCAT-3, SAC, BESS, and BSI-18. Since the aim of this study was to investigate changes in plasma p-tau181 in the acute post-injury phase of SRC, all time points subsequent to the initial post-injury (0–48 h) time point were excluded from the present study.

#### Imaging protocol (THINC cohort only)

For mTBI patients, clinical CT and research MRI were performed within 48 h of injury. A standardized MRI protocol was used, which included DTI, T2^*^-weighted imaging, T2-Fluid-Attenuated Inversion Recovery (FLAIR), high-resolution 3D-T1, dynamic susceptibility contrast perfusion-weighted imaging, and post-contrast T1- and T2-FLAIR. The detailed methods of the imaging protocol have been previously reported (30–32). The MRI findings were classified into MRI−positive and MRI−negative groupings. MRI findings were deemed positive based on the presence of any of the following indications: dural enhancement of meninges, microbleed, epidural hematoma, subdural hematoma, subarachnoid hemorrhage, contusion, intracerebral hemorrhage, or intraventricular hemorrhage.

### Blood sample processing and analysis

Venipuncture blood was collected within 48 h of injury in plastic dipotassium ethylenediaminetetraacetic acid tubes, immediately placed on ice, centrifuged (15 min, 1,500*g*, room temperature), and stored at −80°C until analysis. Plasma protein quantifications were analyzed using the Single Molecule Array p-tau181 V2 Advantage kit (Quanterix, Lexington, MA) for measurement of p-tau181 protein on HD-x Analyzer^TM^. The samples were processed according to the manufacturer's instructions. Researchers were blind to the sample's demographic and clinical information. Samples that reported below the lower limits of quantification (0.338 pg/ml) and had a coefficient of variation higher than 20% were excluded from the analysis. The protein quantification in both cohorts was within the assay range.

### Statistical analysis

Statistical analyses were conducted using SPSS version 28.0.0 (Armonk, NY: IBM Corp.), and GraphPad Prism 8.4 was used to generate graphs (La Jolla, CA: GraphPad Software). Biomarker concentrations underwent natural log-transformation for normality. The log-transformed variables were subsequently used for all analyses. Independent sample *t*-tests, chi-square (χ^2^) tests, and one-way analysis of variance (ANOVA) tests were performed to determine the group differences. The biomarker was presented in median with 25th and 75th percentiles. Receiver operating characteristic (ROC) and area under the curve (AUC) analyses with a 95% confidence interval (CI) were used to evaluate the ability of the p-tau181 protein concentrations to discriminate mTBI patients from UIC and concussed athletes from non-concussed athletes. AUCs and ROCs were also evaluated by the time between injury and blood sampling (0–6 h of injury, 7–18 h of injury, 19–24 h of injury, and >25 h of injury). Statistical significance was determined at a *P*-value of < 0.05 in all analyses.

## Results

### THINC cohort

The demographic and clinical characteristics of the participants are presented in Table 1. The study participants were 18–96 years old (45.50 ± 17.25 years). There were no significant differences in age [*t* (72.58) = 1.61, *P* = 0.056], sex [χ^2^ (1) = 0.59, *P* = 0.440], or race [χ^2^ (4) = 1.83, *P* = 0.767] between the mTBI and UIC groups. In mTBI patients, the mean time from injury to blood draw was 17.49 h (SD = 2.87).

**Table 1:** Demographic and clinical information of the THINC cohort.

	**UIC (*n =* 30)**	**mTBI (*n =* 288)**	**Significance**
**Age, years, mean (SD)**	48.27 (7.51)	45.47 (18.20)	*t* (72.58) = 1.61, *P* = 0.056
**Sex**, ***N*** **(%)**			
Men	20 (66.7)	211 (73.3)	χ^2^ (1) = 0.59, *P* = 0.440
Women	10 (33.3)	77 (26.7)	
**Race**, ***N*** **(%)**			
White	19 (63.3)	201 (69.8)	χ^2^ (4) = 1.83, *P* = 0.767
Black or African American	10 (33.3)	71 (24.7)	
Asian	1 (3.3)	8 (2.8)	
Multiple race	0 (0.0)	3 (1.0)	
Others/unknown	0 (0.0)	5 (1.7)	
**Ethnicity**, ***N*** **(%)**			
Latino or Hispanic	2 (6.7)	57 (19.9)	χ^2^ (1) = 3.12, *P* = 0.077
Not Latino or Hispanic	28 (93.3)	230 (80.1)	
**Injury mechanism**, ***N*** **(%)**			
Acceleration/deceleration	NA	39 (13.6)	
Direct impact- head	NA	64 (22.4)	
Fall-ground floor	NA	83 (29.0)	
Fall from height >1 m	NA	37 (12.9)	
**NSI, mean (SD)**	NA	17.95 (14.32)	
**GCS**, ***N*** **(%)**			
15		212 (73.6)	
14		43 (14.9)	
13		9 (3.1)	
≤12		15 (5.2)	
Unknown		9 (3.1)	
**CT positive**, ***N*** **(%)**	NA	81 (29.0)	
**MRI positive**, ***N*** **(%)**	NA	159 (56.2)	

UIC, uninjured controls; mTBI, mild traumatic brain injury; SD, standard deviation, NSI, neurobehavioral symptom inventory; GCS, Glasgow Coma Scale; CT, computed tomography; MRI, magnetic resonance imaging.

**Figure 1:**
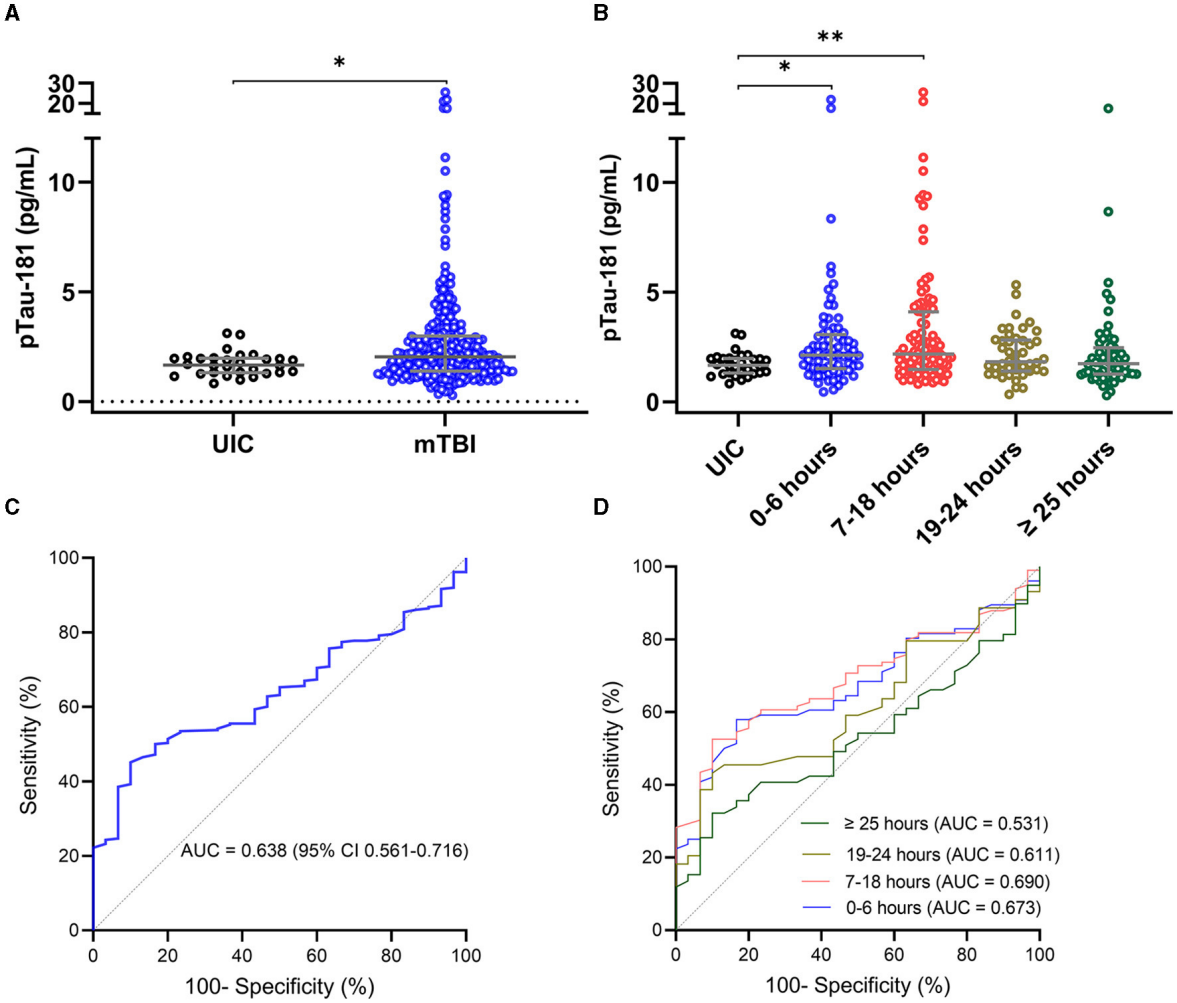
Plasma concentration of p-tau181 in THINC cohort. Plasma p-tau181 concentration is presented in scatter dot plots with the median and 25–75th percentiles in mTBI patients vs. UIC within 48 h of injury **(A)** and by the time of the blood draw **(B)**. Receiver operating characteristic curves and area under the curve (AUC) for p-tau181 in mTBI patients vs. UIC were within 48 h of injury **(C)** and by the time of the blood draw **(D)**. Significance differences are indicated with ^*^P < 0.05 and ^**^P < 0.01. UIC, uninjured controls; mTBI, mild traumatic brain injury.

Plasma p-tau181 concentration was significantly elevated in mTBI patients compared with the UIC (mean difference, 0.28 ln pg/ml; 95%CI, 0.03–0.52 ln pg/ml). The median concentration of p-tau181 in mTBI patients was 0.72 ln pg/ml (0.32–1.10) as compared to 0.51 ln pg/ml (0.26–0.68) in UIC (Figure 1A).

The time stratification of p-tau181 concentration over time is presented in Figure 1B. The average number of hours from injury to blood sampling was 4.6 h for 0–6 h of injury (*n* = 76), 13.4 h for 7–18 h of injury (*n* = 99), 22.0 h for 19–24 h of injury (*n* = 44), and 37.6 h for >25 h of injury (*n* = 59). The median concentration of p-tau181 in mTBI patients was 0.76 ln pg/ml (0.41–1.12) for 0–6 h of injury, 0.77 ln pg/ml (0.39–1.141) for 7–18 h of injury, 0.60 ln pg/ml (0.34–1.04) for 19–24 h of injury, and 0.55 ln pg/ml (0.23–0.90) for >25 h of injury (Figure 1B). ANOVA analysis showed significant group differences for the p-tau181 biomarker (*F*_(4, 303)_ = 4.343; *P* = 0.0020). The levels of p-tau181 were significantly elevated in mTBI patients within 0–6 h of injury (mean difference, −0.30 ln pg/ml; *P* = 0.0435) and 7–18 h of injury (mean difference, −0.42 ln pg/ml; *P* = 0.0049) compared to UIC but not 19–24 h of injury (mean difference, −0.15 ln pg/ml; *P* = 0.3320) and after 25 h of injury (mean difference, −0.08 ln pg/ml; *P* = 0.4188).

The AUC for p-tau181 to discriminate between mTBI patients and UIC was 0.638 (95%CI, 0.561–0.716) within 48 h of injury (Figure 1C). The AUC for p-tau181 to discriminate between mTBI patients and UIC was 0.674 (95%CI, 0.572–0.776, *P* = 0.0055) for 0–6 h of injury, 0.691 (95%CI, 0.599–0.782, *P* = 0.0016) for 17–18 h of injury, 0.612 (95%CI, 0.484–0.739, *P* = 0.1044) for 19–24 h of injury, and 0.532 (95%CI, 0.413–0.651, *P* = 0.6239) for >25 h of injury (Figure 1D).

### Association of plasma p-tau181 with neuroimaging findings

To understand the association of plasma p-tau181 levels with different neuroimaging findings, mTBI patients were classified based on their clinical CT and research MRI groups: (1) CT−/MRI− (negative CT and MRI; *n* = 111), (2) CT+/MRI+ (positive CT and MRI; *n* = 74), and (3) CT−/MRI+ (negative CT and positive MRI; *n* = 89) compared with UIC (*n* = 30). Fourteen cases were excluded due to missing CT or MRI data. The median concentration of p-tau181 in patients with CT−/MRI− was 0.59 ln pg/ml (0.23–0.97); CT+/MRI+ was 0.76 ln pg/ml (0.27–1.30); CT−/MRI+ was 0.83 ln pg/ml (0.51–1.13); and in UIC was 0.51 ln pg/ml (0.26–0.68).

**Figure 2:**
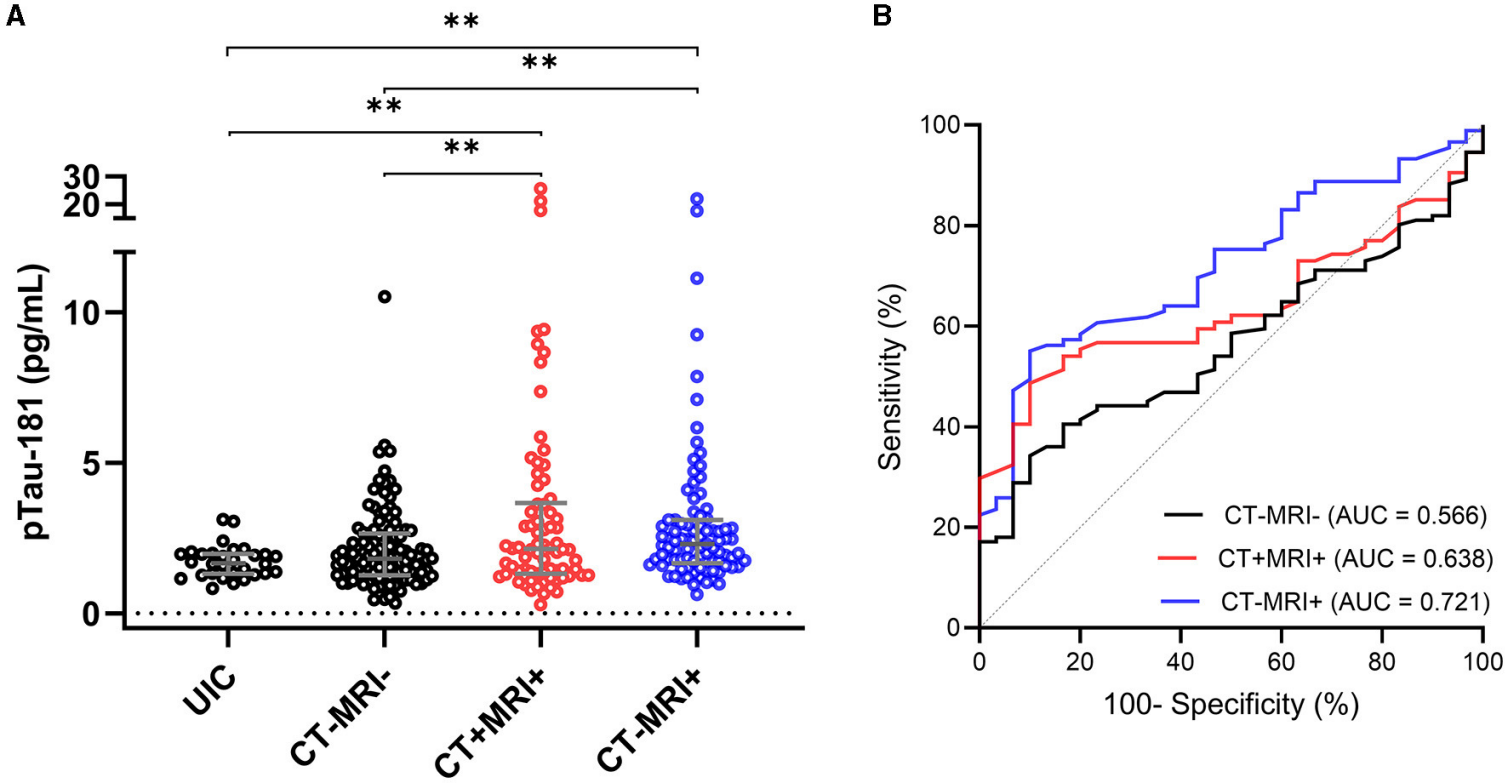
Plasma concentration of p-tau181 by neuroimaging groups in the THINC cohort. Plasma p-tau181 concentration is presented in scatter dot plots with the median and 25–75th percentiles in UIC vs. mTBI patients within 48 h of injury with or without neuroimaging findings **(A)**. Receiver operating characteristic curves and area under the curves (AUC) for p-tau181 in mTBI patients with and without neuroimaging findings vs. UIC **(B)**. Significance differences are indicated with ^*^P < 0.05 and ^**^P < 0.01. UIC, uninjured controls; mTBI, mild traumatic brain injury; CT, computed tomography; MRI, magnetic resonance imaging.

ANOVA analysis showed a significant group difference for the p-tau181 biomarker (*F*_(3, 300)_ = 5.913; *P* = 0.0006) (Figure 2A). The level of p-tau181 was significantly higher in patients with CT+/MRI+ (mean difference, −0.37, *P* = 0.0042) and those with CT−/MRI+ (mean difference, −0.40, *P* = 0.0032) compared with UIC but not in patients with CT−/MRI− (mean difference, −0.11, *P* = 0.1431).

AUC analyses were used to determine the ability of biomarkers at the acute time point of mTBI to discriminate mTBI patients with and without neuroimaging findings from UIC (Figure 2B). The AUC for p-tau181 to discriminate between mTBI patients with CT−/MRI− and UIC was 0.566 (95%CI, 0.467–0.665, *P* = 0.2688). In stratifying positive imaging findings, the p-tau181 biomarker had AUC of 0.639 (95%CI, 0.535–0.742, *P* = 0.0274) for discriminating patients with positive CT and MRI from UIC. Plasma p-tau181 had an AUC of 0.721 (95%CI, 0.627–0.816, *P* = 0.0003) for distinguishing positive MRI and negative CT patients from UIC.

### CARE cohort

This cohort included 133 participants between the ages of 17 and 23 years (mean = 18.76 [SD = 1.13]); the majority were males [*n* = 104 (78.2%)]. The concussion and control groups did not significantly differ on age [*t* (131) = −1.23, *P* = 0.220], sex [χ^2^ (1) = 0.06, *P* = 0.808], race [χ^2^ (5) = 3.93, *P* = 0.559], or ethnicity [χ^2^ (2) = 0.74, *P* = 0.690]. The concussion group had significantly higher SCAT symptom severity [*t* (114.57) = 11.43, *P* < 0.001], BESS total score [*t* (39.01) = 2.82, *P* = 0.008], and BSI-18 global severity index scores [*t* (80.54) = 4.85, *P* < 0.001] compared to the non-concussed athletes. The SAC total score was significantly lower in the concussion group compared to the non-concussed athletes [*t* (117) = −2.46, *P* = 0.015]. The demographic and clinical characteristics of the participants are presented in Table 2.

**Table 2:** Demographic and clinical characteristics of CARE cohort.

	**Non-concussion (*n =* 21)**	**Concussion (*n =* 112)**	**Significance**
**Age, mean (SD), year**	19.14 (1.32)	18.79 (1.16)	*t* (131) = −1.23, *P* = 0.220
**Sex**, ***N*** **(%)**			
Men	16 (76.2)	88 (78.6)	χ^2^ (1) = 0.06, *P* = 0.808
Women	5 (23.8)	24 (21.4)	
**Race, No. (%)**			
White	14 (66.7)	74 (66.1)	χ^2^ (5) = 3.93, *P* = 0.559
Black	6 (28.6)	18 (16.1)	
Asian	1 (4.8)	8 (7.1)	
Hawaiian or Pacific Islander	0 (0.0)	2 (1.8)	
Multiple	0 (0.0)	8 (7.1)	
Unknown/not reported	0 (0.0)	2 (1.8)	
**Ethnicity**, ***N*** **(%)**			
Non-Hispanic	19 (90.5)	94 (83.9)	χ^2^ (2) = 0.74, *P* = 0.690
Hispanic	1 (4.8)	6 (5.4)	
Unknown/not reported	1 (4.8)	12 (10.7)	
**Years of sport participants, mean (SD)**	10.95 (4.71)	10.36 (4.16)	*t* (108) = −0.57, *P* = 0.574
**Weight, mean (SD), kg**	83.99 (19.12)	85.63 (20.63)	*t* (130) = 0.34, *P* = 0.736
**Height, mean (SD), cm**	178.53 (8.04)	180.29 (10.44)	*t* (130) = 0.74, *P* = 0.465
**Sport, No. (%)**			
Football	9 (42.9)	44 (39.3)	χ^2^ (9) = 7.83, *P* = 0.551
Ice hockey	0 (0.0)	6 (5.4)	
Lacrosse	2 (9.5)	9 (8.0)	
Rugby	2 (9.5)	8 (7.1)	
Soccer	6 (28.6)	17 (15.2)	
Volleyball	0 (0.0)	1 (0.9)	
Water polo	0 (0.0)	1 (0.9)	
Wrestling	2 (9.5)	6 (5.4)	
Cheerleading	0 (0.0)	1 (0.9)	
Not reported	0 (0.0)	19 (17.0)	
**SCAT symptom severity score, mean (SD)**	3.57 (4.97)	28.77 (19.20)	*t* (114.57) = 11.43, *P* < 0.001
**BESS total score, mean (SD)**	11.24 (5.82)	15.51 (7.93)	*t* (39.01) = 2.82, *P =* 0.008
**BSI global severity index, mean (SD)**	1.43 (2.25)	5.09 (5.63)	*t* (80.54) = 4.85, *P* < 0.001

CARE, Concussion Assessment Research and Education; SD, standard deviation, SCAT, Sport Concussion Assessment Tool; SAC, Standardized Assessment of Concussion; BESS, Balance Error Scoring System; BSI, Brief Symptom Inventory.

**Figure 3:**
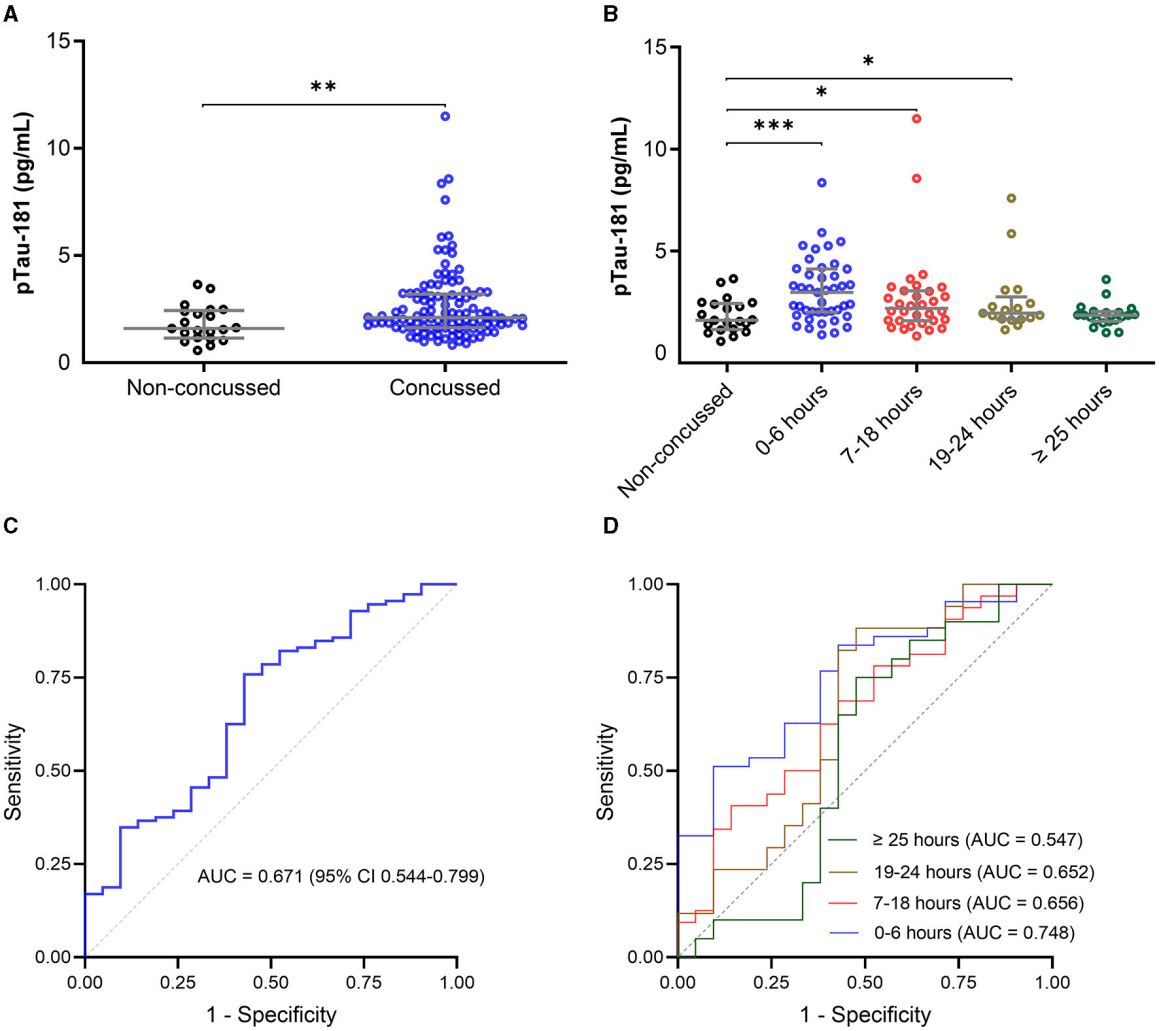
Plasma concentration of p-tau181 in the CARE cohort. The plasma p-tau181 concentration is presented in scatter dot plots with the median and 25–75th percentiles in concussed athletes vs. non-concussed athletes within 48 h of injury **(A)** and by the time of the blood draw **(B)**. Receiver operating characteristic curves and area under the curves (AUC) for p-tau181 in concussed athletes vs. non-concussed athletes within 48 h of injury **(C)** and by the time of the blood draw **(D)**. Significance differences are indicated with ^*^P < 0.05, ^**^P < 0.01, and ^***^P < 0.001.

The plasma p-tau181 concentration was significantly elevated in concussed athletes compared with non-concussed athletes (mean difference, −0.36 ln pg/ml; 95%CI, −0.59 to −0.12 ln pg/ml). The median concentration of p-tau181 in concussed athletes was 0.75 ln pg/ml (0.50–1.17), compared to 0.47 ln pg/ml (0.14–0.89) in non-concussed athletes (Figure 3A).

The time stratification of p-tau181 concentration over time is presented in Figure 3B. The average number of hours from injury to blood sampling was 2.6 h for 0–6 h of injury (*n* = 43), 15.6 h for 7–18 h of injury (*n* = 32), 21.4 h for 19–24 h of injury (*n* = 17), and 39.8 h for >25 h of injury (*n* = 20). The median concentration of p-tau181 in concussed athletes was 1.12 ln pg/ml (0.69–1.42) for 0–6 h of injury, 0.78 ln pg/ml (0.45–1.11) for 7–18 h of injury, 0.67 ln pg/ml (0.50–1.01) for 19–24 h of injury, and 0.62 ln pg/ml (0.48–0.71) for >25 h of injury (Figure 3B). ANOVA analysis showed a significant group difference for the p-tau181 biomarker (*F*_(4, 128)_ = 4.727; *P* = 0.0014). The level of p-tau181 was significantly elevated in concussed athletes within 0–6 h of injury (mean difference, −0.51 ln pg/ml; *P* = 0.0003), 7–18 h of injury (mean difference, −0.32 ln pg/ml; *P* = 0.0218), and 19–24 h of injury (mean difference, −0.30 ln pg/ml; *P* = 0.0407)[-4pt] compared to non-concussed athletes, but not after 25 h of injury (mean difference, −0.12 ln pg/ml; *P* = 0.2326).

The AUC for p-tau181 to discriminate between concussed and non-concussed athletes was 0.671 (95%CI, 0.544–0.799) within 48 h of injury (Figure 3C). The AUC for p-tau181 to discriminate between concussed and non-concussed athletes was 0.748 (95%CI, 0.626–0.870, *P* = 0.0013) for 0–6 h of injury, 0.656 (95%CI, 0.505–0.807, *P* = 0.0562) for 17–18 h of injury, 0.653 (95%CI, 0.476–0.829, *P* = 0.1096) for 19–24 h of injury, and 0.548 (95%CI, 0.362–0.733, *P* = 0.6019) for >25 h of injury (Figure 3D).

## Discussion

To the best of our knowledge, this is the first prospective study to examine plasma p-tau181 levels in acute mTBI patients and athletes with SRC. We used an ultrasensitive digital enzyme-linked immunosorbent assay to quantify p-tau181 protein levels using discovery kits. The levels of plasma p-tau181 were significantly elevated in mTBI patients within 48 h of injury compared to UIC. Notably, in mTBI patients, plasma p-tau181 levels were significantly elevated within 18 h of injury and returned to similar levels with UIC after 24 h of injury. Plasma p-tau181 levels were also significantly elevated in concussed athletes compared with non-concussed athletes with the concentration of p-tau181 reaching a transient peak within 6 h. In addition, our study showed that concentrations of p-tau181 were highest in ED patients with neuroimaging findings. Among mTBI patients, the levels of plasma p-tau181 were significantly higher in patients with positive neuroimaging (either CT+/MRI+, *n* = 74 or CT−/MRI+, *n* = 89) compared to mTBI patients with negative neuroimaging (CT−/MRI−, *n* = 111) findings and UIC. The AUC for discriminating CT−/MRI+ from UIC showed modest (0.72) discrimination. Comparable discrimination within this same cohort was previously reported for GFAP (AUC of 0.74) in distinguishing patients with mTBI who were CT−/MRI+ from mTBI patients who were both CT− and MRI−, suggesting its potential utility in determining patients with mTBI with subtle injuries detected only through MRI (33). These findings indicate that plasma p-tau181 concentrations likely relate to brain injury, with the highest levels in patients with neuroimaging evidence of injury, and that similar to GFAP, it may aid in the detection of more subtle injuries detected only through MRI, an important finding since the presence of subtle injuries not observed on CT, including traumatic axonal injury, increases risks for neurological symptoms (34, 35).

To evaluate the temporal effect of p-tau181 released or accumulated in the peripheral circulation, we stratified the groups based on the time between injury and blood collection sampling: 0–6 h of injury, 7–18 h of injury, 19–24 h of injury, and >25 h of injury. The concentration of p-tau181 reached a peak within 18 h of injury in the THINC cohort. Within the CARE cohort, the concentration of p-tau181 reached a transient peak within 6 h and then quickly diminished over 48 h. In a prior study within the CARE cohort, GFAP, NFL, UCH-L1, and total tau were also found to be most elevated in concussed athletes at the acute post-injury time point compared to control athletes and were considerably lower at 24–48 h after injury (36). The AUCs at the initial post-injury time point ranged from 0.53 to 0.68 for the individual markers with the highest AUC being for GFAP (0.68), followed by UCH-L1 (0.66), NFL (0.56), and finally total tau (0.55), and with AUCs of 0.70–0.71 for the combination of GFAP and UCH-L1 and 0.72 for the 4-plex of biomarkers (36). In the present study, the p-tau181 AUC for discriminating concussed athletes from non-concussed athletes was highest (0.75) between 0 and 6 h. This finding suggests that, when assessed within 6 h of injury, p-tau181 may outperform GFAP, UCH-L1, NFL, and total tau as a diagnostic marker of acute SRC in athletes. Plasma p-tau181 levels within 6 h of sport-related injury showed similar diagnostic accuracy to GFAP, NFL, and UCH-L1 in discriminating military service academy cadets with combat-related concussion from uninjured cadets at the acute post-injury phase (<6 h) (37). Together, these findings suggest that plasma p-tau181 may provide better results in the diagnosis of SRC than total tau in the acute phase and that the combination of p-tau181 with GFAP, NFL, and UCH-L1 may potentially enhance the diagnostic accuracy of a multi-marker panel for mTBI/concussion.

The tau phosphorylation site at threonine 231 has been investigated in all severities of TBI, but p-tau181 has not. A previous study showed that plasma p-tau231 was elevated in mild-to-severe TBI patients at acute and chronic time points compared to healthy control populations (20). Additionally, plasma p-tau231 was significantly higher in TBI patients with a positive CT and predicted poor outcomes at 6 months of follow-up (20). Here, p-tau181 levels were significantly elevated in CT−positive mTBI patients compared to CT−negative mTBI patients and UIC. Among the 200 CT−negative mTBI patients, 89 had findings on MRI. Levels of p-tau181 were significantly elevated in CT−/MRI+ mTBI patients compared to imaging-negative (CT−/MRI−) mTBI patients and UIC. P-tau181 levels did not significantly differ between imaging-negative (CT−/MRI−) mTBI patients and UIC. Pooled cohort and stratifying analyses were used to determine the ability of p-tau181 to discriminate mTBI patients with and without neuroimaging findings from UIC and showed the highest discrimination accuracy in the neuroimaging stratified analysis for discriminating CT−/MRI+ mTBI patients from UIC. Specifically, p-tau181 showed a modest (AUC = 0.72) ability to discriminate mTBI patients with subtle injuries detected on MRI only from UIC, suggesting its potential utility in detecting more subtle injuries not observed on CT. This finding is in agreement with a recent study showing elevated p-tau181 in retired athletes with a history of repetitive concussions compared to controls and that an elevation in plasma p-tau181 is associated with brain abnormalities detected with MRI (26). Acute axonal injury and microstructural alterations of white matter are hallmark features of mTBI, but their subtle nature can often be detected only in research settings. In fact, it is estimated that up to 25–40% of CT−negative patients are MRI−positive, which increases the likelihood of developing neurological symptoms, which is linked to long-term and poor recovery (34, 38–41). Similarly, DTI findings within the CARE cohort are related to delayed recovery. Since advanced imaging modalities are not widely available in emergency room settings, alternative biomarkers sensitive to the subtle nature of these injuries are critically needed to inform clinical decisions. These findings suggest that p-tau181 may be a potential marker for the subtle injury characteristic of mTBI and SRC, detectable only through advanced MRI imaging that is not readily accessible outside of the research setting.

In summary, our results suggest that p-tau181 may have a potential role as a diagnostic marker of mTBI in patients with MRI findings as well as SRC. The findings presented here suggest the need for additional studies that include multiple biomarkers to determine the combination of biomarkers that will improve the diagnostic accuracy of mTBI/SRC. Future studies involving larger cohorts are needed, which would allow for analyses to delineate each biomarker's diagnostic and prognostic value to formulate an optimal biomarker panel to identify neuroimaging findings and predict clinical outcomes. Our findings indicate that blood-based biomarkers of p-tau181 may aid in clinical decisions in the acute period and may ultimately assist in developing targeted-personalized management following mTBI/SRC.

Although this is the first study to investigate the diagnostic utility of plasma p-tau181 in mTBI and SRC, our findings may be constrained by the discovery nature of the assay kits and cross-sectional design. The finding of the temporal effect of plasma p-tau181 is constrained by a cross-sectional design. Despite this limitation, these preliminary findings show that plasma p-tau181 is elevated in acute mTBI and SRC. Plasma p-tau181 shows some promise as an early, promising biomarker of acute mTBI and SRC and requires additional studies to determine its value in relation to neuroimaging findings and clinical recovery. Issues related to the specificity of this assay remain critical to understand. This is one of the first studies in younger cohorts, so additional studies using this assay are required to understand the content of these findings and their future implications through more longitudinal studies in the future. Furthermore, future studies are needed to investigate the impact of sex on the clinical interpretation of plasma p-tau181 concentrations in acute mTBI and SRC. In AD, several studies have shown that higher levels of tau biomarkers are more strongly associated with clinical AD and disease progression in women than in men; however, it is unknown whether these sex differences apply to plasma p-tau181 (42–45). Moreover, plasma p-tau181 levels may be impacted by multiple factors, including race and ethnicity, which were not included in our models in this study. This study is also limited by the fact that MRI scans were not performed for UIC as it is possible that performing such scans could show relevant findings that may have been important to this study since, in theory, one or more of the controls could have had one or more small lesions that could have been detected with advanced imaging, and these lesions may exist/persist in the absence of any symptoms or known history of neurological injury or disease. An additional limitation is that “baseline” imaging for the mTBI cases prior to incurring injury was not available. Finally, follow-up analysis was not performed to assess the relationship between temporal changes in p-tau181 and injury resolution as determined by imaging over time.

## Data availability statement

The original contributions presented in the study are included in the article/supplementary material, further inquiries can be directed to the corresponding author.

## Ethics statement

The studies involving human participants were reviewed and approved by the Institutional Review Boards of the National Institutes of Health, Uniformed Services University of the Health Sciences, MedStar Washington Hospital Center, Johns Hopkins Suburban Hospital Privacy Board, Medical College of Wisconsin, and the Human Research Protection Office at the US Army Medical Research and Material Command. The patients/participants provided their written informed consent to participate in this study.

## Author contributions

SPB, TM, CCG, PP, LL, MAM, and JMG designed and conceptualized the study. RV contributed to the analysis. RV, CD, SM, TBM, CL, SPB, TM, CCG, DH, JH, KLC, GM, JJ, KG, JPM, AB, SD, SR, LDN, PP, CT, LL, MAM, and JMG interpreted the data. RV, CD, and JMG drafted the manuscript for intellectual content. SM, TBM, CL, SPB, TM, CCG, DH, JH, KLC, GM, JJ, KG, JPM, AB, SD, SR, LDN, PP, CT, LL, and MAM revised the manuscript for intellectual content. RV and CL contributed to the acquisition. All authors contributed to the article and approved the submitted version.
